# Temporal distribution of peak running demands relative to match minutes in elite football

**DOI:** 10.5114/biolsport.2022.110745

**Published:** 2021-12-30

**Authors:** Bradley Thoseby, Andrew D. Govus, Anthea C. Clarke, Kane J. Middleton, Ben J. Dascombe

**Affiliations:** 1 Sport and Exercise Science, School of Allied Health, Human Services and Sport, La Trobe University; 2 High-Performance Department, Melbourne City Football Club, Melbourne, Australia; 3 Applied Sport Science and Exercise Testing Lab, School of Environmental and Life Sciences, University of Newcastle, Australia

**Keywords:** Team sports, Soccer, Distribution, Peak match intensities, Peak running intensities

## Abstract

The peak match running demands of football (soccer) have been quantified across time durations of 1–10 min, however, little is known as to when the peak match running demands occur within match play. Data were collected from 44 elite footballers, across 68 fixtures (Files = 413, mean ± SD; 11 ± 8 observations per player, range; 1–33), with peak match running demands quantified for each playing half at ten incremental rolling average durations (1 min rolling averages, 2 min rolling averages, etc.). Data were assessed if players completed the full match. Three measures of running performance were assessed total distance (TD), high-speed distance (> 19.8 km · h^-1^) (HSD) and average acceleration (AveAcc)], with the in-game commencement time of the peak running demands recorded. Descriptive statistics and normality were calculated for each rolling average duration, with the self-containment of shorter rolling average epochs within longer epochs also assessed (e.g. Do the 1 min peak running demands occur within the 10 min peak running demands). Peak TD and AveAcc demands occurred early in each half (median time = 7–17 min and 6–16 min, respectively). Conversely, peak HSD covered was uniformly distributed (Skewness = 0–0.5, Kurtosis = 1.7–2.0). There were low-moderate levels of self-containment for each peak match running period (10–51%), dependent upon metric. Peak match running demands for TD and AveAcc occurred at similar stages of a match where TD and acceleration volumes are typically greatest, whereas peak HSD demands appeared more unpredictable. These timings may help inform training prescriptions in preparation of athletes for competition.

## INTRODUCTION

Wearable technology allows for the quantification of the physical match demands of football (soccer), aiding in the prescription and monitoring of athlete training loads [1]. Historically, the physical demands of match-play have been quantified through reporting the absolute distance covered, both overall and within various speed thresholds [2,3]. Conventionally, match demands are reported as a function of the entire match or broken down into smaller periods (e.g. between halves or 5–15 minute blocks), in an effort to provide insight into within-match fluctuations in the absolute physical outputs [4,5,6,7]. Previous researchers have identified that the absolute total distance (TD) and high-speed distance (HSD) covered tends to decrease as a match progresses, with physical demands greatest in the first 15 min and lowest in the final 15 minutes of a match [7,8,9]. Similar findings have also been reported for acceleration counts, with the number of accelerations (> 2 m · s^-2^) being significantly higher in the 0–15 and 15–30 minute periods than the 60–75 and 75–90 minute periods [10]. Such information regarding temporal shifts in performance and match demands are useful to practitioners in gauging and monitoring athlete performance, while also helping to provide insight into acute fatigue and potentially guiding pacing strategies. However, while informative, the use of such data to inform training practices may be limited, with the use of absolute or relative total match data likely to under-prepare athletes for shorter periods of higher intensity efforts during different match periods. As such, alternate methods that quantify the intensity of match play and identify the most physically demanding periods of match play have been developed, providing practitioners additional data that informs training prescription [11,12].

Practitioners have begun to quantify the peak match running demands of football across durations significantly shorter than previously reported time periods (e.g. 15, 45 or 90-min) 13,14,15]. The quantification of the peak match running demands involves the identification of the most physically demanding periods of a match across pre-determined window durations of 1–10 minutes [12,14,16]. The use of a rolling average window (e.g. 0–1 min, 0.1–1.1 min) has demonstrated to be superior in quantifying peak match running demands when compared to discrete time periods (e.g. 0–1 min, 1–2 min, etc.), with discrete periods underestimating both peak total and HSD by -7–10% and -12–25%, respectively, across the 1–10 minute window durations [11,12,17]. However, little is known as to the temporal distribution of peak match running demands, i.e. when the peak match running demands occur during match-play, with there being over 54,000 instances throughout a match where the peak 1 min running demands may occur (if using 10Hz GPS devices) [18,19]. Taken together, peak match running demands reflect the greatest physical demands that are required of an athlete throughout match-play. Importantly, the typical window durations associated with peak match running demands (1–10 min) better align with those associated with football-based conditioning drills than the discrete 15, 45 or 90-min windows [20].

Across recent years, the peak match running demands of football have initially been investigated, with new data exploring the impact of contextual factors, such as competitional and positional differences, beginning to emerge [15,21,22]. This additional context surrounding peak match running demands has proven useful in ensuring that prescribed training drills simulate the demands typical of match-play and provide an adequate stimulus for preparing athletes for competition. However, while temporal changes in the absolute running demands of match play have been observed [2,10,23,24,25], the temporal distribution of peak match running demands remain to be thoroughly investigated. To date, a single study has attempted to assess the temporal distribution of peak match running demands, however, the study in question used a categorised the timings of peak match running demands into discrete 15 min bins, as opposed to using a continuous time scale, from which the temporal distribution of peak match running demands could be more accurately determined [26]. Such information may help to identify the more specific periods of play when peak match running demands occur, helping to inform practitioners around how to structure training sessions accordingly to best replicate matches.

Furthermore, with peak match running demands typically quantified over time durations of varying lengths (1–10 min), it is possible that peak match running demands of shorter durations may occur within longer peak match running durations. For example, the peak running demands observed for a 5 min duration may self-contain the peak running demands of 1–4 min, which would indicate that athletes are infrequently required to perform at peak match intensities. Conversely, if peak running demands of shorter durations are not self-contained within longer peak running durations, this would indicate athletes are frequently required to perform at each peak match intensities. Currently, the self-containment of peak match running demands is yet to be reported on, with this information likely proving useful to coaches in ensuring the number of drills replicating match demands within a training session is appropriate in preparing athletes for competition. Therefore, the current study aims to identify the temporal distribution of when peak match running demands occur during competitive football match-play, and to quantify the self-containment levels of peak match running demands.

## MATERIALS AND METHODS

Activity profiles of elite football players were measured during 68 competitive A-League matches, spanning three seasons (2015–2018) (2015/16 Season = 25 matches, 2016/17 Season = 15 matches, 2017/18 = 26 matches). Match GPS files were downloaded for 44 elite football players from the one club with a total of 413 individual match observations (mean ± SD; 11 ± 8 observations per player, range; 1–33). As this study aims to identify when peak match demands occur within each half rather than the magnitude of these demands, all positional groups were combined, with data from players who played less than 90 minutes not included for analysis in order to avoid any artificial skewing of results. Goalkeepers were excluded due to their positional demands not being reflective of the group. “Prior to collection of data, ethical approval was attained from La Trobe University (HREC#: 18056).

### Activity Profile

Data were collected during match play using 18 Hz portable GPS units (STATSports, Northern Ireland), secured between the shoulder blades of the athlete using a custom-made harness, with data downloaded post-match using proprietary software (STATSports, Northern Ireland). These GPS devices have shown strong validity and reliability in the measurement of locomotor speeds across varying velocities (Bias < 2.11%; CV < 2.91%; ICC = 0.95–0.98) [27,28]. Statistical software (R Studio, v1.2.5033) was used to analyse exported raw GPS files (inclusive of added time) using custom functions. The raw exported speed trace was filtered using a 4^th^ order one-way Butterworth filter with a cut-off frequency of 1 Hz. Individual data points exceeding a running speed of 10 m · s^-1^ were deemed as erroneous and replaced with zero values. Similarly, acceleration/deceleration magnitudes greater than excess of 6 m · s^-2^ were classified as technical errors and replaced with zero values. The effect of these replacements was deemed negligible (< 0.01% of data points replaced), due to the method used to quantify the peak match running demands.

The measures chosen for the assessment of running intensity were total TD covered, HSD covered (> 19.8 km · h^-1^) and average acceleration (AveAcc). Both TD and HSD covered were expressed relative to unit of time (m · min^-1^), with AveAcc calculated as per established methods where the absolute values of all accelerations and decelerations are summated and averaged over a defined duration (m · s^-2^) [29]. While it is recognised that the amalgamation of both accelerations and decelerations into a singular metric may conceal the underlying mechanism of load, the use of this metric provides greater insight into the overall intensity of an activity [14]. These values were then mapped to in-game accumulative time, recorded at the commencement of the peak running duration. For example, a peak 1 minute running duration occurring between the 40^th^ and 41^st^ minute would be recorded as commencing at the 40^th^ minute. The mapping of peak running demands to the minute in which they commenced was used, as opposed to when they finished, to combat the potential artificial skewing of longer duration epochs and allow for direct comparisons between various rolling average durations. As additional time was included in the analyses, it is possible for the commencement of peak running durations to occur later than the 45^th^ minute.

### Statistical Analysis

Statistical analyses were conducted using R Studio statistical programming software (v1.2.5033, R Core Development Team, Vienna). Descriptive statistics of means, standard deviations, median values, and interquartile ranges were calculated for each intensity period, with normality at each intensity period calculated using skewness and kurtosis measures. Skewness and kurtosis were calculated using the ‘moments’ package [30]. The magnitude of skewness was quantified using the following descriptors: approximately symmetric (-0.5 < S_KP_ < 0.5), moderately skewed (-1 < S_KP_ < -0.5 or 0.5 < S_KP_ < 1) or highly skewed (S_KP_ < -1 or 1 < S_KP_). Descriptors for kurtosis were assigned as: mesokurtic (β_2_ = 3), platykurtic (β_2_ < 3) or leptokurtic (β_2_ > 3). A larger skewness value (in either direction) demonstrates that peak match running demands predominantly occur at either the start or end of a match half. Subsequently, the kurtosis value would indicate how heavy-tailed or light-tailed the distribution is, with a high value indicating the presence of outliers, i.e. peak running demands occurring at either the start or end of a match (depending on skewness) are atypical, with a low kurtosis value indicating that there are minimal/no outliers to the distribution. Additionally, the temporal self-containment of shorter peak match intensity windows within longer windows was assessed by determining whether the entirety of a peak running period occurred within a longer window. The self-containment window is defined as the entirety of a time duration across which peak match running demands occur for a given rolling average duration. For example, if the peak 1 min period was observed between the 40^th^ and 41^st^ minute and the peak 5 min period was observed between the 38^th^ and 43^rd^ minute, then the peak 1 min period would be recorded as being self-contained with the peak 5 min period. The relative proportion of occurrences of a shorter peak match intensity duration within each longer self-containment window is described as a percentage of total occurrences using the following novel qualitative descriptors: *very low* (< 30%), *low* (30–40%), *moderate* (40–50%), *high* (50–60%) and *very high* (> 60%).

## RESULTS

**FIG. 1:**
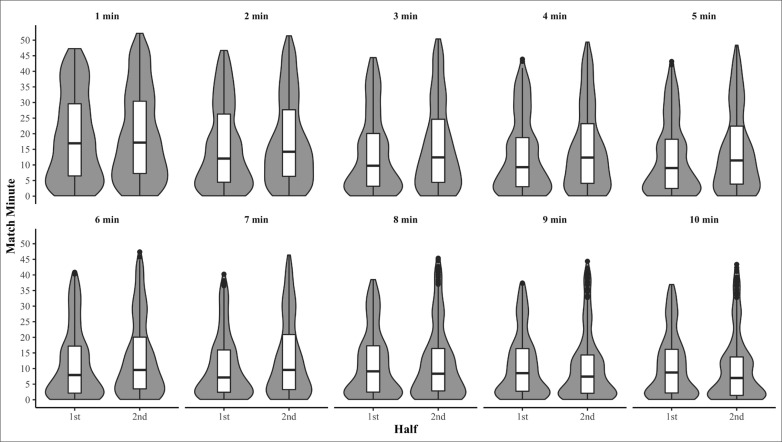
Within-half violin and box plots of when peak relative total distance covered commenced for moving average durations of 1-10 minutes.

**FIG. 2:**
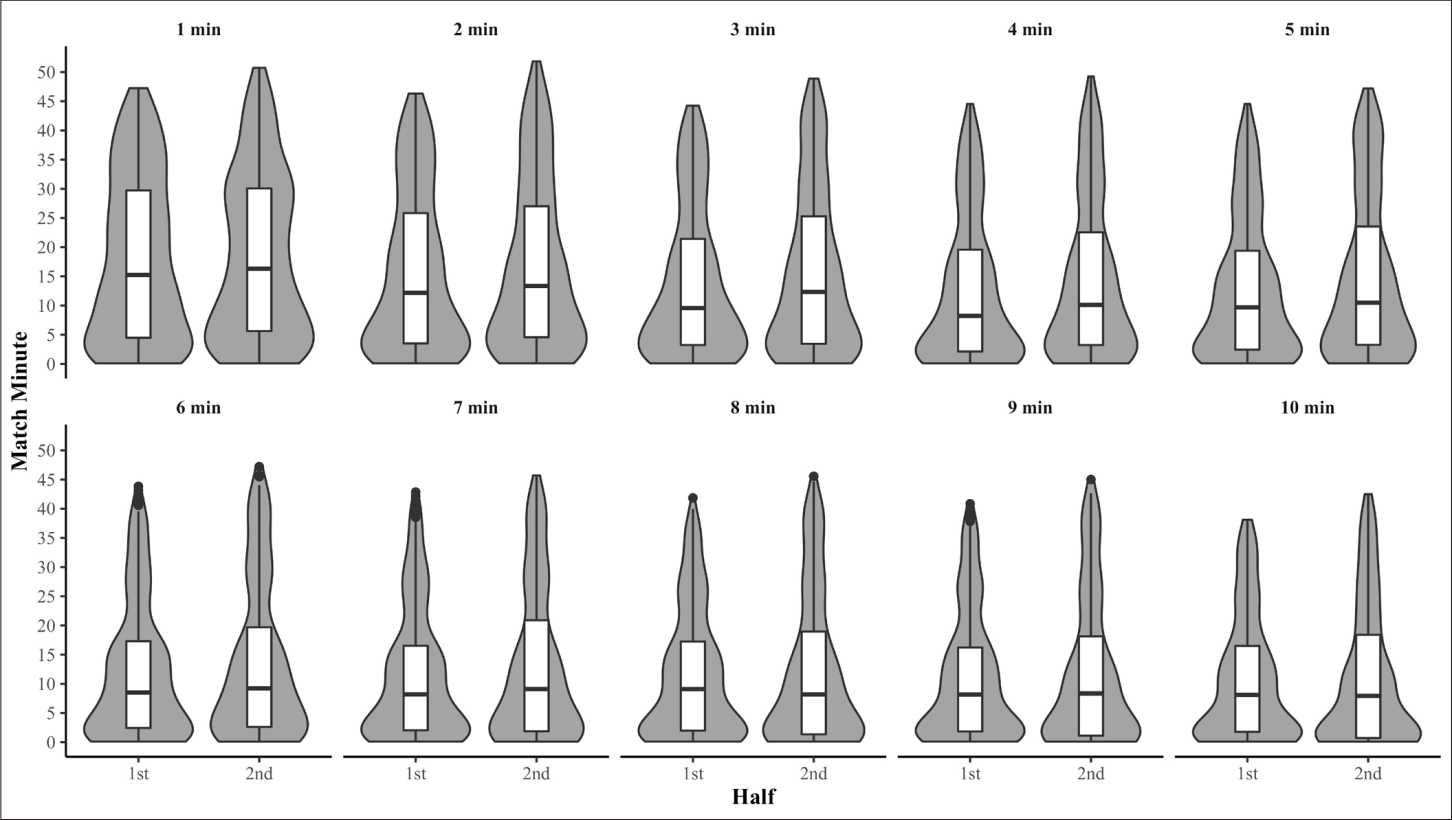
Within-half violin and box plots of when peak average acceleration demands commenced for moving average durations of 1-10 minutes.

**FIG. 3:**
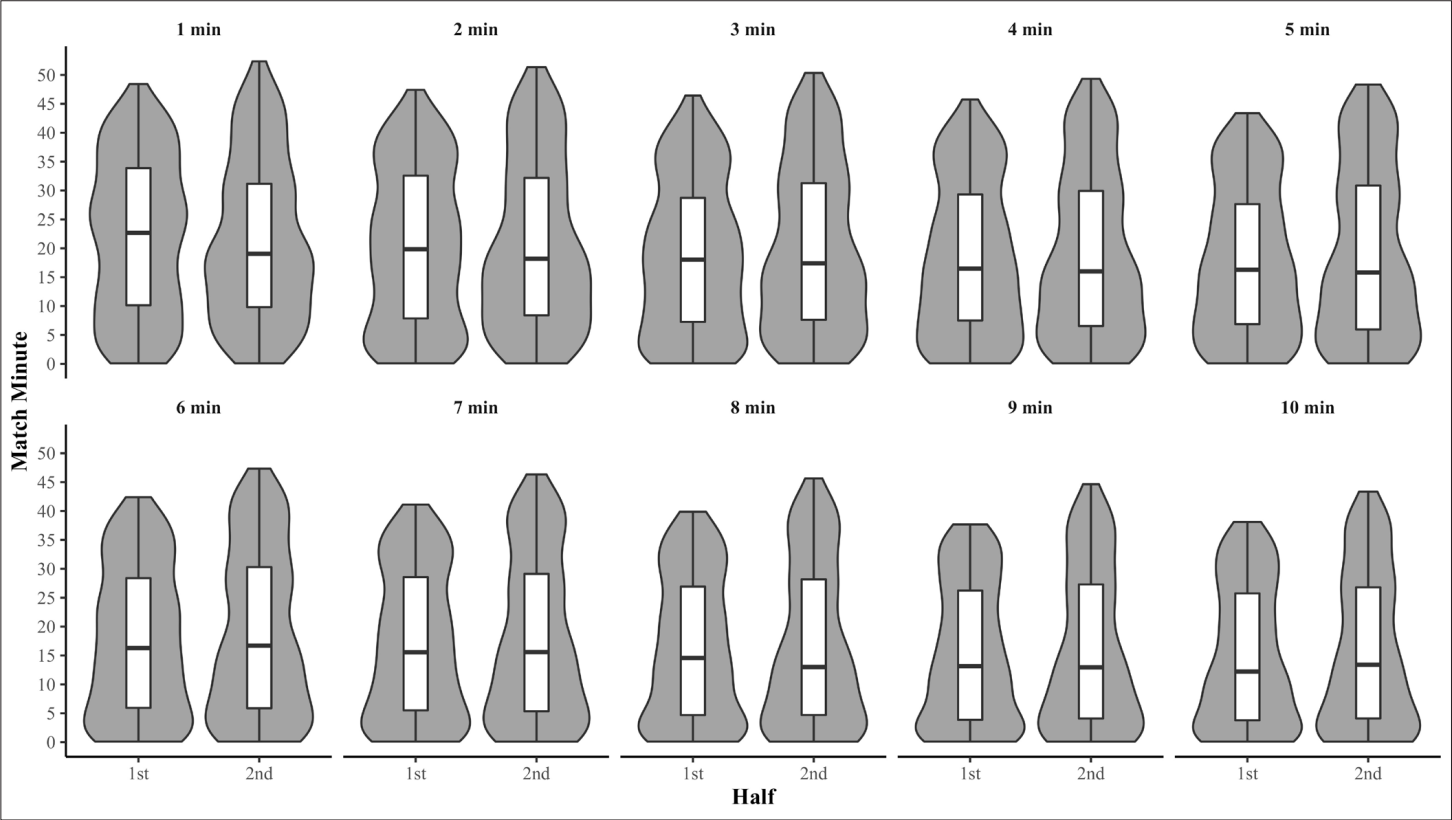
Within-half violin and box plots of when peak relative high-speed distance covered commenced for moving average durations of 1-10 minutes.

Raw distribution descriptive statistics of when peak match running demands occurred are presented in Table 1. Peak match running demands for relative TD (Figure 1) and AveAcc (Figure 2) were moderately to highly right skewed in both the first and second halves, for the majority of intensity periods (Skewness = 0.7–1.2 and 0.6–1, respectively) demonstrating the peak match running demands of TD and AveAcc typical occur early within a half. Conversely, peak running demands of relative HSD (Figure 3) were mostly uniformly distributed across each half for all intensity periods (Skewness = 0–0.5, Kurtosis = 1.7–2.0). The temporal self-containment of peak match intensity periods are presented in Table 2, with the total distance and average acceleration peak match running demands displaying *low* to *high levels of* relative self-containment (32–47% and 30–51%, respectively), indicating peak running demands of a shorter duration typically coincide with those of longer durations. Further, the self-containment of high-speed distance peak running demands demonstrated a larger spread, with very *low* to *moderate* (9–49%) levels of self-containment reported which indicates a large proportion of shorter peak running demands occur irrespective of longer peak running demands.

**TABLE 1:** Raw distribution descriptive statistics of when peak match running intensities occurred during match-play across moving average durations of 1–10 minutes. Data is presented as match minutes of when peak running demands commenced. Skewness and kurtosis are presented as raw statistical values.

Metric Variable		Half	Moving Average Duration									
			1 min	2 min	3 min	4 min	5 min	6 min	7 min	8 min	9 min	10 min
**Relative Total Distance (m.min^-1^)**	Mean ± SD	1st	19 ± 14	16 ± 13	14 ± 13	13 ± 12	12 ± 12	12 ± 11	11 ± 10	11 ± 11	11 ± 10	11 ± 10
		2nd	19 ± 14	18 ± 14	16 ± 14	15 ± 13	14 ± 13	13 ± 12	13 ± 12	12 ± 12	11 ± 11	10 ± 11

	Median	1st	17	12	10	9	9	8	7	9	9	9
		2nd	17	14	12	12	11	10	10	8	7	7

	IQR (Lower-Upper)	1st	23 (7–30)	22 (5–26)	17 (3–20)	16 (3–19)	16 (3–18)	15 (2–17)	14 (2–16)	15 (2–17)	14 (3–16)	14 (2–16)
		2nd	23 (7–30)	21 (6–28)	20 (4–25)	19 (4–23)	19 (4–22)	17 (4–20)	18 (3–21)	14 (3–16)	12 (2–14)	12 (1–14)

	Skewness	1st	0.4	0.7	0.8	0.8	0.8	0.9	1.1	0.8	0.9	0.9
		2nd	0.5	0.7	0.8	0.8	0.8	0.9	0.9	1.1	1.2	1.2

	Kurtosis	1st	1.9	2.2	2.5	2.4	2.6	2.8	3.3	2.6	2.8	2.7
		2nd	2.1	2.4	2.6	2.6	2.6	2.8	2.7	3.3	3.8	3.7

**Relative High-Speed Distance (m.min^-1^)**	Mean ± SD	1st	22 ± 13	20 ± 14	19 ± 13	18 ± 13	18 ± 13	18 ± 13	17 ± 13	16 ± 12	15 ± 12	15 ± 12
		2nd	22 ± 14	21 ± 14	20 ± 14	19 ± 14	19 ± 15	19 ± 14	18 ± 14	17 ± 14	16 ± 13	16 ± 13

	Median	1st	23	20	18	17	16	16	16	15	13	12
		2nd	19	18	17	16	16	17	16	13	13	13

	IQR (Lower-Upper)	1st	24 (10–34)	25 (8–33)	22 (7–29)	22 (8–29)	21 (7–28)	22 (6–28)	23 (6–29)	22 (5–27)	22 (4–26)	22 (4–26)
		2nd	21 (10–31)	24 (8–32)	24 (8–31)	23 (7–30)	25 (6–31)	24 (6–30)	24 (5–29)	24 (5–28)	23 (4–27)	23 (4–27)

	Skewness	1st	0.0	0.1	0.2	0.3	0.3	0.2	0.2	0.3	0.3	0.4
		2nd	0.4	0.4	0.4	0.4	0.4	0.3	0.4	0.5	0.5	0.4

	Kurtosis	1st	1.8	1.8	1.9	1.9	1.9	1.8	1.7	1.8	1.8	1.8
		2nd	2.1	2.0	2.0	2.0	1.8	1.8	1.9	1.9	1.9	1.9

**Average Acceleration (m.s*-2*)**	Mean ± SD	1st	18 ± 14	16 ± 14	14 ± 13	13 ± 12	13 ± 12	12 ± 11	11 ± 11	11 ± 11	11 ± 11	11 ± 11
		2nd	18 ± 14	17 ± 14	16 ± 14	15 ± 13	15 ± 14	14 ± 13	13 ± 13	12 ± 13	12 ± 12	11 ± 12

	Median	1st	15	12	10	8	10	9	8	9	8	8
		2nd	16	13	12	10	10	9	9	8	8	8

	IQR (Lower-Upper)	1st	25 (5–30)	22 (4–26)	18 (3–21)	18 (2–20)	17 (2–19)	15 (2–17)	15 (2–17)	15 (2–17)	14 (2–16)	15 (2–17)
		2nd	24 (6–30)	23 (5–27)	22 (3–25)	20 (3–23)	21 (3–24)	17 (3–20)	19 (2–21)	18 (1–19)	17 (1–18)	18 (1–18)

	Skewness	1st	0.4	0.6	0.8	0.9	0.8	0.9	1.0	0.9	1.0	0.9
		2nd	0.4	0.7	0.7	0.8	0.8	0.9	0.9	1.0	1.0	1.0

	Kurtosis	1st	1.9	2.1	2.5	2.6	2.6	2.9	3.0	2.7	2.9	2.8
		2nd	2.0	2.3	2.4	2.5	2.3	2.6	2.6	2.8	2.8	2.8

**TABLE 2:** Relative (%) level of self-containment of peak match running intensities within the self-containment windows

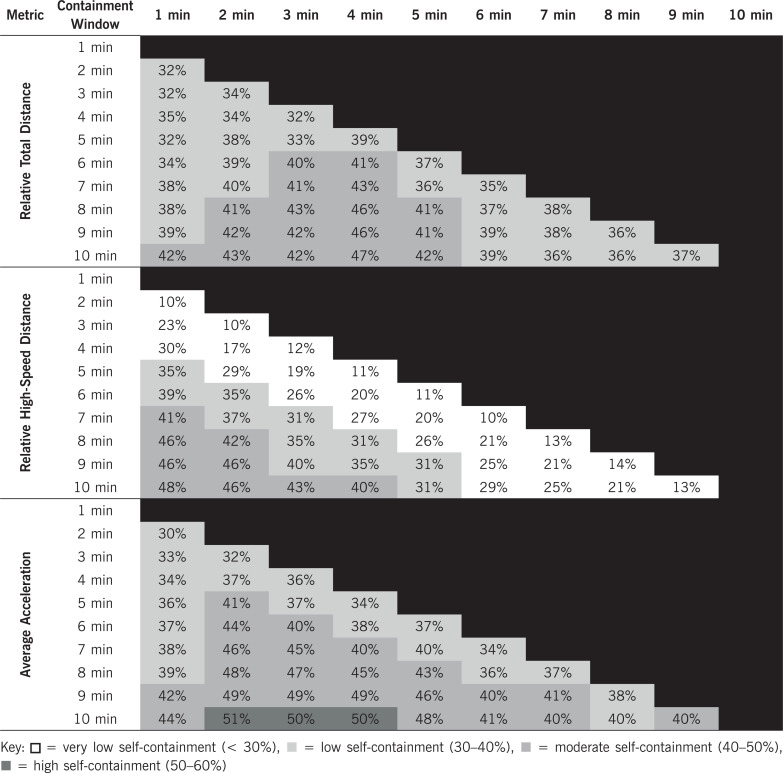

## DISCUSSION

The current study aimed to determine the temporal distribution of when peak match running demands typically occur during elite football matches and secondly, to elucidate the self-containment of match running demands. The primary findings indicated that peak match running demands for relative TD and AveAcc occurred early in each half for all moving average epochs (median time = 7–17 min and 6–16 min, respectively). Separately, the peak relative HSD demands were more evenly distributed across each half (S_KP_ = 0.0–0.5, β_2_ = 1.7–2.1). These findings give insight to when peak running demands typically occur during match halves, while also providing further context for structuring training sessions to appropriately simulate competition demands. Additionally, the current study is the first to assess the temporal self-containment of shorter peak match running periods within longer windows, identifying that less than 55% of shorter peak match intensity periods occur within longer durations. The *very low* to *moderate* levels of self-containment amongst peak match running periods highlights the need for athletes to regularly perform at peak intensities for varying lengths of time. This knowledge may further aid in the design and structure of training drills and sessions through the implementation of numerous drills across a session aimed at replicating peak match demands.

Using discrete time periods (e.g. 0–15 min), temporal reductions in physical output have been observed across both halves and match entirety, with the first 15 minutes of each half requiring the greatest absolute running demands [4,5,6,7,9]. While literature reporting on the AveAcc demands is limited, similar observations have been made for acceleration counts (> 2 m · s^-2^), with the number of accelerations performed in the final 30 minutes of a match significantly less than first [10]. The present study observed similar findings, whereby majority of the peak match running demands of relative TD and AveAcc, irrespective of rolling average window, occurred within the first 17 minutes of each half. These findings are also similar to those previously reported on peak match running demands across a 1 min duration, with peak total distance covered and acceleration counts found to occur predominantly in the first 15 min of match-play [26]. The mechanistic properties behind the declines in physical output across a match, with regard to both volume and intensity reflect factors such as acute fatigue, team tactics and score line [3], with athletes often implementing pacing strategies in an attempt to attenuate the reduction in physical performance [23,31,32]. The apparent alignment of the highest match running volumes, peak match running demands and acceleration counts in the early stages of a half reflects a positive pacing strategy. With coaches aiming to establish superiority in the first 15 minutes of a match [33], the increased physical output observed in this period is likely reflective of players enacting the tactical plans of coaches, with subsequent anticipatory feedback post-this period modulating running performance to ensure successful completion of the match (Tucker, 2009). Additionally, with high-intensity efforts linked to crucial match periods, it is possible that athletes modulate total distances covered to ensure they are able to maintain HSD covered when needed, as evidenced by peak running demands of TD being heavily skewed and peak highspeed demands being more evenly distributed [34,35].

External contextual factors may also contribute to the distribution of match activities in players, with changes reported in the effective playing time in football demonstrating an increase in match interruptions and greater dead ball time during the latter stages of a match [24]. For example, data from the German Bundesliga identified that ball in play time accounted for ~66% of total match time during the first 15 minutes of a match half, but only ~56% in the final 15 minutes [9]. This reduction corresponded to a significant increase in distance covered while walking and a subsequent decrease in physical intensities in the latter stages of a match. Therefore, it appears that the opportunity for uninterrupted match-play decreases in the latter stages of a half, across extended window durations, which may help explain a converse increase in low-intensity activity. Taken together, this may explain why the majority of TD and AveAcc peak match running demands occurred across lengthening window durations occurred in the early stages of each half. As such, both the TD and AveAcc peak match running demands were likely to coincide with longer periods of uninterrupted match-play, which practitioners should consider when structuring the constraints of game-based training drills targeting physical conditioning in order to maximise the ball in time.

Furthermore, the current data demonstrated that the HSD peak match running demands were uniformly distributed across each half (S_KP_ = 0.0–0.5, β_2_ = 1.7–2.1). These results are similar to past research that has assessed temporal trends in total HSD where reductions were present only in the final 15 minutes of a match, with the preceding 15 minute periods requiring similar demands [7]. Additionally, athlete sprint requirements have previously been shown to be evenly distributed across a match, with slightly higher sprint demands in the first 15 min of each half and in the second half when compared to the first [36]. High-intensity efforts have been closely linked to crucial match events, such as creating or defending goal scoring opportunities [34,35], which may be the case in the shorter window lengths reported in the current study. Analyses of goal timing during international level football matches demonstrates that most goals are scored in the final 15 minutes of a match, with goals scored in all preceding 15 minute periods being equally distributed [37,38]. With goal scoring opportunities occurring frequently and randomly across a match, athletes must continue to perform at higher intensities to maximise offence and defensive success which likely explains the uniform distribution of peak match demands of HSD. It is important to note that the peak match running demands of competition have shown to differ based on factors such as micro-cycle length and positional group [39,40], however, due to cluster size constraints, the impact of these factors on the temporal distribution of peak match running demands were unable to be conducted in the current study and should be further investigated.

Understanding the self-containment of peak match running demands during match-play may help inform the design of training sessions and variety of drills aimed at simulating match-play. For each physical performance metric, there was increasing levels of self-containment observed for shorter peak match intensity windows as the self-containment window duration lengthened. However, differing levels of self-containment were observed at each self-containment window as peak match intensity window increased. Self-containment increased for HSD peak match periods as the self-containment window duration increased and peak running duration decreased, while for TD and AveAcc, self-containment increased as self-containment window duration increased and peak running duration increased, until 2–4 minutes, before then decreasing. Overall, there were *very low* to *moderate* levels of self-containment (< 55%), which likely represents that peak periods occur frequently across a match. Further, with the decreasing margin of containment possible as peak match intensity window increases, i.e. there are more time points for which a 1 minute period can be fully contained within a 10 minute window compared to a 9 minute period, there is the possibility of increasing overlap, as opposed to containment as peak match intensity duration increases. Additionally, for HSD it appears that shorter peak running durations may dictate when longer peak match running periods occur, while for TD and AveAcc it appears that shorter durations (1–4 min) occur more frequently in isolation during match-play. Hence, athletes are regular required to perform at peak intensities for varying lengths of time, with the *very low* to *moderate* levels of self-containment suggesting athletes should be exposed to match simulation drills of varying durations within a single session.

The current study provides insight into the temporal distribution of peak match running demands in elite football, which can help provide greater insight how to appropriately structure training sessions to prepare athletes for the most physically demanding phases of match-play. Exposing athletes to peak match running demands in the initial stages of training will largely replicate what is experienced during match-play and may help coaches to improve athlete’s ability to perform under fatigue after performing at peak match intensities. Conversely, the structuring of match simulation drills at the end of a session, may help develop the capacity of athletes to continue to perform at peak intensities while under fatigue. Further, the unpredictable nature of peak HSD demands requires athletes to perform at such intensities across all stages of a match, which should also be reflected in training. With coaches possibly aiming to expose athletes to peak running demands across a spectrum of durations, information surrounding self-containment may aid coaches in the prescription of drills aimed at replicating the peak match running demands for a number of durations within a single drill. Alternatively, understanding that < 55% of the time peak match running periods do not overlap may warrant athlete exposure to match simulation drills across various durations. As such, tailoring of drills throughout a session to promote HSD efforts may be a more conducive way to replicate match demands, rather than targeting this metric in isolation.

This study is the first to report on the temporal distribution of peak match running demands during competitive football match-play. This information provides further important context to coaches regarding when the most physically demanding periods of match-play occur, helping to provide ecological validity to match-simulation training practices. While it has previously been reported that greater running volume (TD) and acceleration demands occurs in the first 15 minutes, this is the first data to show that the greatest peak demands also occur during this period. Conversely, while high-speed running demands have also shown to be greatest in the first 15 minutes before reducing across the match, the peak match running demands for HSD appear uniformly distributed across each half. With practitioners aiming to prepare athletes for the rigors of competition, the present study provides insight on when peak match running demands typically occur which may prove useful in the planning and structure of training sessions.

## Disclosure Statement

No potential conflict of interest was reported by the authors.
